# Optofluidic fabrication for 3D-shaped particles

**DOI:** 10.1038/ncomms7976

**Published:** 2015-04-23

**Authors:** Kevin S. Paulsen, Dino Di Carlo, Aram J. Chung

**Affiliations:** 1 Department of Mechanical, Aerospace, and Nuclear Engineering, Rensselaer Polytechnic Institute, 110 8th Street, Troy, New York 12180, USA; 2 Department of Bioengineering, University of California, Los Angeles, California 90095, USA

## Abstract

Complex three-dimensional (3D)-shaped particles could play unique roles in biotechnology, structural mechanics and self-assembly. Current methods of fabricating 3D-shaped particles such as 3D printing, injection moulding or photolithography are limited because of low-resolution, low-throughput or complicated/expensive procedures. Here, we present a novel method called optofluidic fabrication for the generation of complex 3D-shaped polymer particles based on two coupled processes: inertial flow shaping and ultraviolet (UV) light polymerization. Pillars within fluidic platforms are used to deterministically deform photosensitive precursor fluid streams. The channels are then illuminated with patterned UV light to polymerize the photosensitive fluid, creating particles with multi-scale 3D geometries. The fundamental advantages of optofluidic fabrication include high-resolution, multi-scalability, dynamic tunability, simple operation and great potential for bulk fabrication with full automation. Through different combinations of pillar configurations, flow rates and UV light patterns, an infinite set of 3D-shaped particles is available, and a variety are demonstrated.

## Abstract (web summary)

The current methods of fabricating three-dimensional particles include photolithography, layer-by-layer printing and several others. Here, Paulsen *et al*. demonstrate an optofluidic approach, whereby masked ultraviolet light is illuminated on photosensitive fluids whose cross-sections are shaped by fluid inertia.

## Graphical abstract

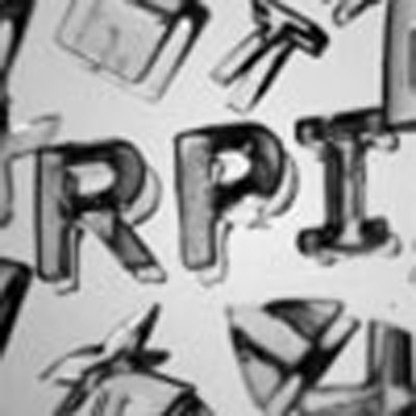

Small particles in nature are often spherical, such that their surface energy is minimized. Non-spherical shapes, however, can provide unique functionalities that are not always available from solely spherical shapes. For example, particles with three-dimensional (3D) morphologies have been shown to behave differently under electrical stimulation^1^, allow for higher packing density^2^ and alter optical characteristics^3^. Thus, non-spherical particles can be of great use for applications such as self-assembly building blocks^4^, structural materials^5^, photonics^6^, pharmaceutics^7^, drug delivery^8^ and tissue engineering^9^. However, based on current manufacturing methods, it is difficult to fabricate large quantities of multi-scale, complex and high-resolution particles in a fast and efficient manner^4^,^10^. One of the promising technologies capable of producing shaped particles is 3D printing^11^. Although this technology allows for the creation of complex geometries from a wide range of materials, including plastics^12^,^13^, ceramics^14^,^15^, metal^16^,^17^ and even human tissue^18^,^19^, 3D printing is still limited by its slow layer-by-layer printing process and low resolution (typical minimum printable size is approximately 45 μm (ref. 20)). As another alternative, injection moulding is a common manufacturing method capable of creating complex geometries of plastics, ceramics or metals^21^, but particle shapes cannot be easily reconfigured since new costly moulds are required for any design modifications. A successful moulding technology called PRINT^22^,^23^ is used for the bulk fabrication of micro- and nano-scale particles. This technology allows for control of size, shape, composition and surface chemistry of particles, but shapes are limited by an imprint mould. Recently, it has been demonstrated that microfluidic droplet-based flow lithography^24^,^25^,^26^,^27^ can be used for continuous and high-throughput fabrication of micrometre-scale objects. These methods, however, are restricted to creation of only spherical and round-shaped objects as they rely on surface tension between immiscible fluids. Other new microfluidic-based methods termed stop flow lithography (SFL)^28^,^29^ and optofluidic maskless lithography (OFML)^30^,^31^ have shown potential for fabricating high-resolution particles in a continuous process. In short, patterned ultraviolet (UV) light is illuminated on a ‘static' microfluidic channel containing UV reactive fluid. The techniques showed success in creating various shapes, sizes and multifunctional particles^32^,^33^,^34^; however, the methods are inherently limited because they depend on two-dimensional (2D) extrusions for particle formation. Through light interference patterns^35^, greyscale lithography^36^ or layer-by-layer polymerization^37^, limited 3D shapes have been demonstrated, although those approaches were still based on the creation of 2D extruded shapes.

A new method presented here, referred to as optofluidic fabrication, allows for the creation of complex 3D-shaped particles in a simple, scalable and high-resolution manner. Optofluidic fabrication is based on two coupled processes: (i) deterministic inertial flow shaping in fluidic channels and (ii) UV polymerization of photosensitive fluid streams. In traditional microfluidics, fluid inertia is neglected because of small-length scales and low flow rates, resulting in near zero Reynolds number flows (that is, Stokes flow; *Re*<<1). Reynolds number (*Re*) is a dimensionless parameter representing the ratio between inertial forces and viscous forces that exist in a fluid (*Re=ρUL*_c_*/μ*; *ρ* is the fluid density, *U* is the fluid velocity, *L*_c_ is the channel characteristic length and *μ* is the fluid dynamic viscosity). However, the assumption of negligible fluid inertia in microfluidics often becomes invalid when flow involves higher velocities. At finite Reynolds numbers (*Re*=10–100), distinct inertial effects play a significant and useful role in microchannels. For instance, in a curved channel, fluid elements near the channel centre travel faster than those near the channel walls, creating vortices with a vorticity axis parallel to the main flow known as Dean flow^38^,^39^,^40^. As another example, recently it was reported that localized secondary flows can be created by placing structures such as pillars and columns in straight channels^41^,^42^,^43^,^44^. This localized secondary flow differs from Dean flow, which perturbs the entire flow field in the channel. Pillars strategically positioned inside of microchannels can be used to create deterministic localized vortices and additive complex flow shapes depending on the pillar size, placement, number of sequential pillars and flow conditions^43^,^44^.

Here, we present a novel method termed ‘optofluidic fabrication' that utilizes the localized secondary flow from pillars placed in fluidic channels to shape the flow cross-section of UV polymerizable fluid streams. In short, complex 3D-shaped particles can be formed at the intersection of vertically patterned UV light and horizontally shaped UV reactive fluid, as shown in Fig. 1. By altering flow conditions and UV light patterns/conditions, an infinite set of shaped particles can be created. Using optofluidic fabrication, we demonstrate rapid fabrication of complex shaped particles, introducing a programmable and continuous fabrication paradigm for non-spherical structures.

## Results

### 3D particle generation

**Figure 1:**
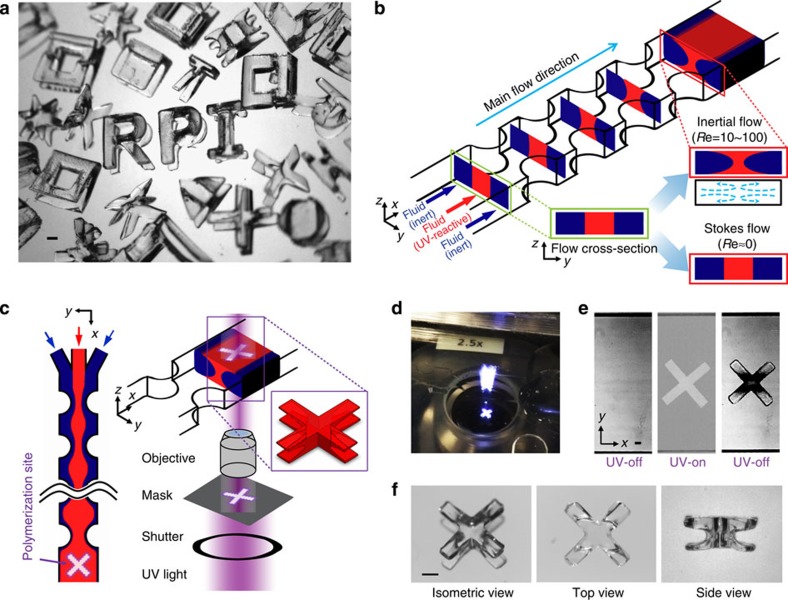
Optofluidic fabrication of 3D particles. (**a**) Examples of 3D-shaped particles. (**b**) Design and operating principles for inertial flow deformation. A photosensitive core fluid stream (red) and inert sheath fluid streams (blue) enter a rectangular channel containing half-pillars located along the channel sidewalls. Local vortices created near the pillars deform the fluid cross-section, resulting in an altered flow cross-sectional shape downstream. (**c**) A schematic showing the particle generation process. Once flow deformation is achieved, the flow is stopped, the channel is illuminated with ‘X'-shaped UV light, and a predicted particle shape is shown. (**d**,**e**) Experimental demonstration of 3D-shaped particle generation. From left to right: far field view of the UV polymerization, channel imaged before UV illumination, patterned (‘X') UV light illumination and the resulting polymerized 3D-shaped particle. (**f**) Images of the fabricated particle imaged outside of the channel. All scale bars represent 500 μm.

The fundamental principle of creating an infinite set of 3D-shaped particles relies on combining (i) geometry-induced flow deformation by fluid inertia and (ii) orthogonal masked UV polymerization (see Fig. 1). First, to create deterministic flow deformations, we used poly(dimethylsiloxane) (PDMS) channels consisting of three inlets, one outlet and a series of pillars strategically arranged within the channel. Two sheath fluid streams, poly(ethylene glycol) diacrylate (PEG-DA), entered along the channel sides, and sandwiched a photosensitive core fluid stream, PEG-DA with photoinitiator (2,2-dimethoxy-2-phenylacetophenone (DMPA)) that was injected in the centre of the channel. For the inertial flow case, as the fluid streams passed a pillar, interactions between fluid inertia and the pillar geometry causes permanent deformation of the cross-sectional shape of the fluid streams, creating fore and aft asymmetry in the channel (see Fig. 1b). As flow passes sequential pillars, each pillar induces a net secondary flow, adding to the overall flow deformation. Conversely, for Stokes flow, the reversibility of the flow deformation^45^ leads to no net fluid cross-sectional changes (Fig. 1b), preventing flow shaping.

For the second fabrication step, UV polymerization, the inertially deformed flow is quickly stopped (<1 s) and exposed to patterned UV light for 1.5 s (Fig. 1c–e) to form a particle. The particle is generated at the intersection of the shaped photosensitive fluid stream and patterned UV light. For example, as shown in Fig. 1c–f, by illuminating ‘X' patterned UV light, a particle with an ‘X' shape from the top view and an ‘I' shape from side view can be fabricated (see Supplementary Movie 1). By employing various flow and light conditions, an infinite set of particle shapes is possible.

### Numerical analysis on geometry-induced inertial flow

**Figure 2:**
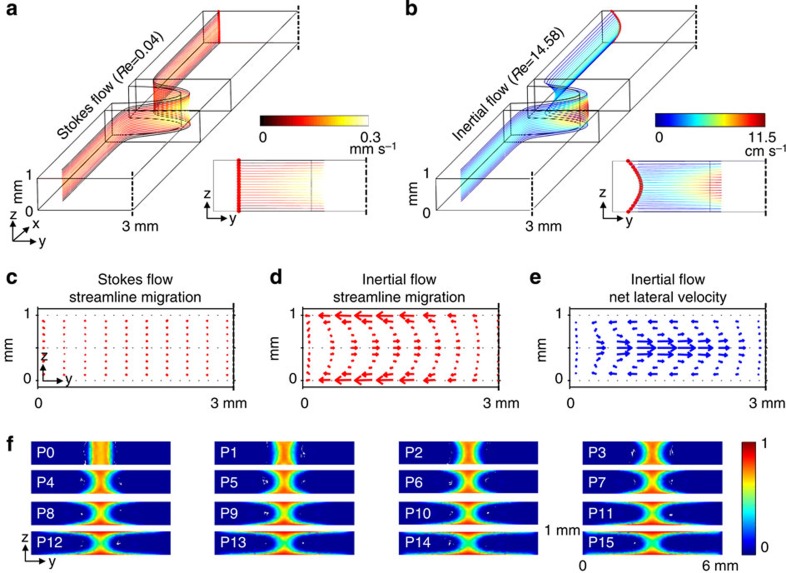
Numerical analysis of the inertial flow. (**a**) Streamlines in a side-pillar channel for Stokes flow (*Re*=0.04) and (**b**) inertial flow (*Re*=14.58) after fluid elements pass one side pillar. Note that only half of the channel is simulated because of channel symmetry. (**c**) Vector plots of net streamline displacement for Stokes flow and for (**d**) inertial flow. (**e**) Net lateral velocity plot for inertial flow. (**f**) Convection-diffusion solution showing the expected flow deformation after passing 15 sequential side pillars at *Re*=14.58.

To understand and predict the intrinsic inertial flow deformation associated with a pillar sequence, we conducted a finite element-based numerical analysis. We created rectangular channels with the following pillar configurations and dimension: width (*W*) of 6 mm, height (*H*) of 1 mm, pillar spacing (*S*) of 7 mm and pillar diameter (*D*) of 3.83 mm. The channel and pillar dimensions were carefully chosen based on the mode of flow deformation^43^. Because of the high viscosity of the PEG-DA solution (15 mPa s), large channels were employed to exert inertial effects in channels while maintaining reasonable pressure drops. It should be noted that a large channel does not necessarily imply the ability to only create larger particles or structures. As the particle size is determined by the intersection of light and shaped photosensitive fluid, this intersection volume may be made smaller than the channel dimension. In addition, by utilizing UV reactive fluids with lower viscosity and an advanced light setup, channels with smaller dimensions can be used for colloidal-scale particle generation^29^. A single-pillar case was first investigated where one pair of half-cylindrical pillars (referred to as side pillars) are located along the channel sides, as shown in Fig. 2a–e. To compare the effects of fluid inertia on flow deformation, both Stokes and inertial flow cases were simulated. Note that due to channel symmetry, the flow field for only half of the channels was calculated. The complete Navier–Stokes equations were solved to compute the flow field inside of the channels. To examine the amount of fluid deformation caused by a single pair of side pillars, fluid streamlines were traced as flow passed the pair of side pillars. For the Stokes flow case (*Re*=0.04), there was little to no lateral migration of streamlines after passing a pillar (Fig. 2a,c); however, significant lateral streamline migration (Fig. 2b,d) was shown for the inertial case (*Re*=14.58). Figure 2d plots the streamline migration in the *y*-*z* plane for the inertial case, and clearly shows net lateral streamline displacement. To incorporate the time dependence of the streamline migration, net lateral velocities^46^ for the inertial case were also calculated and plotted (see Fig. 2e).

To study how the inertial flow changes as a function of sequential pillar numbers, the flow fields of multi-pillar cases were also solved. Based on the solution from the full Navier–Stokes equation at *Re*=14.58, the convection-diffusion equation was solved for a side-pillar channel with 15 sequential pairs of side pillars. Figure 2f indicates the normalized concentration profiles, clearly showing the core fluid distortion upon passing a series of pillars.

### Experimental demonstration of inertial flow shaping

**Figure 3:**
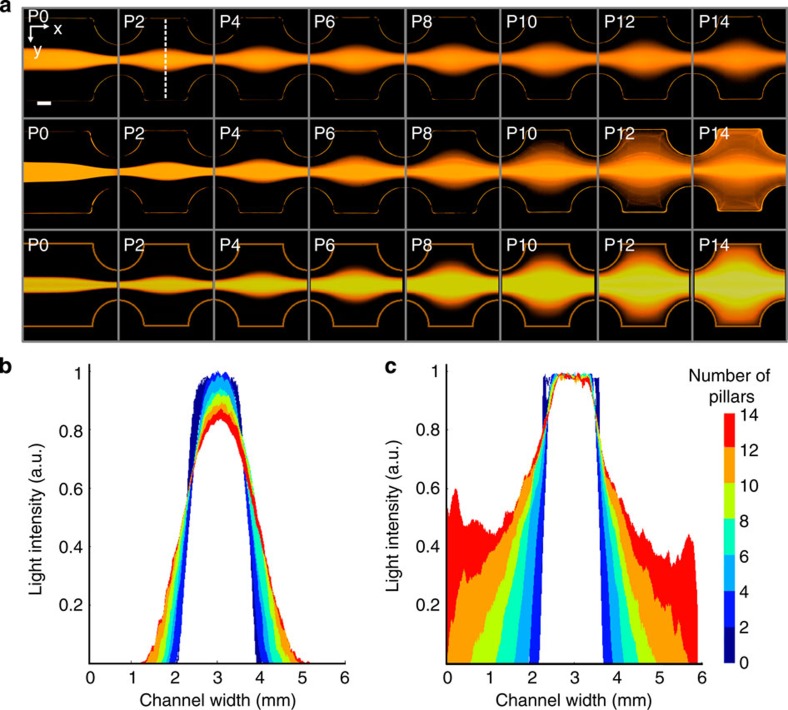
Experimental inertial deformation. (**a**) Core fluid stream dyed with Rhodamine B passes sequential side pillars for a Stokes flow (row 1) and inertial flow (row 2) case. Row 3 shows a numerical simulation for the inertial case of expansion of core fluid in an *x*–*y* slice along the top of the channel (*z*=0.95 mm). All images are false-colour. (**b**) Light intensity variation along a line drawn between pillars (white dashed line in **a**) for the Stokes flow and (**c**) inertial flow case. Scale bar represents 1 mm.

To experimentally verify the fluid deformation predicted by numerical simulations, channels with sequential side pillars were imaged when co-flowing streams of fluorescent and non-fluorescent dye. The core fluid stream was doped with fluorescent dye (Rhodamine B) and examined for both a Stokes flow and inertial flow case (See Fig. 3 and Supplementary Movie 2). Figure 3a, row 1, shows the experimental Stokes flow case (*Re*=0.003) from the channel top view. ‘P0' depicts the channel before passing any pillars, and P‘N' indicates the channel after passing the ‘N'-th pillar. Ideally, for the Stokes flow case, the width of the core fluid should remain unchanged after passing pillars, but the fluorescent dye was diffused into the sheath fluid, causing the core fluid to appear visibly wider. For the inertial case (*Re*=14.58), as shown in Fig. 3a, row 2, the core fluid stream is distorted extensively, causing the fluorescent core stream to widen along the channel top and bottom. As detailed in the previous section, a convection-diffusion equation was solved, and numerical solutions of the inertial case are shown in Fig. 3a, row 3, where an *x*–*y* slice along the top of the channel (at *z*=0.95 mm) is presented, showing very good agreement between the numerical prediction and experiment.

To help quantify the inertial deformation and expansion of the fluorescent core stream, light intensity plots were created. Figure 3b,c describes the change in intensity across the entire width of the channel as a function of the number of sequential pillars. The Stokes flow case (Fig. 3b) shows a slight widening of the fluorescent stream as mentioned above, and the inertial flow case (Fig. 3c) shows significant expansion of the core fluid. The small intensity peaks seen for pillar 14 near the channel walls were caused by the core fluid widening until it reaches the channel walls, and then turning direction as it reaches the walls.

### Flow shaping via pillar configuration

**Figure 4:**
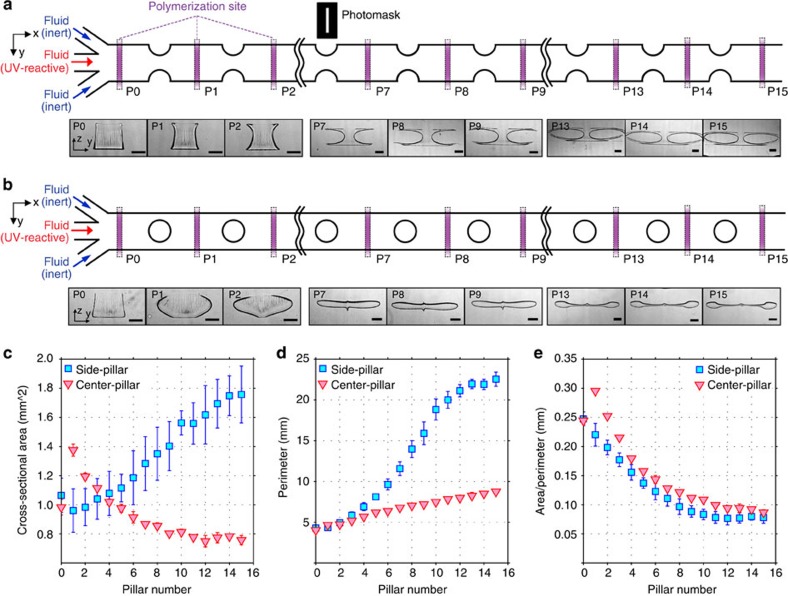
Evolution of flow deformation. Inertial flow deformation depends on the number of sequential pillars and pillar configuration. At a fixed Reynolds number (*Re*=14.58), the cross-sectional shape evolution is shown for (**a**) side-pillar and (**b**) centre-pillar channels through polymerization using a photomask with a thin, rectangular opening. (**c**) Plots of cross-sectional area, (**d**) perimeter and (**e**) particle characteristic length (particle area/particle perimeter) as a function of number of pillars. Error bars represent one standard deviation from the mean (*n*=3; from different experimental setups). All scale bars represent 500 μm.

The fabricated particle shapes are determined by both flow and light conditions. The flow shaping is based on the interactions between fluids and structures (pillars); therefore, by manipulating the lateral placement of pillars in the channel, number of sequential pillars and flow rate (*Re*), we can alter the cross-sectional flow shape to an infinite set of conditions. As a first effort, we investigated the dependence of flow shape on number of pillars. To capture the fluid cross-sectional flow shaping after passing multiple pillars, we polymerized particles after each pillar (see Supplementary Movie 3) using a thin and long, slit-shaped photomask (see Fig. 4a). As shown in Fig. 4a, the evolution of the flow cross-sectional area is clearly captured by the changing particle shape as fluid passes each pillar. We also investigated the pillar configuration dependence by testing a channel possessing a sequence of centre-located pillars (Fig. 4b and Supplementary Movie 3) with identical flow conditions (*Re*=14.58). Similar to the four vortices generated by the side-pillar case, as shown in Fig. 1b), the centre-pillar case creates four vortices with opposite rotational directions, causing the core fluid to be widened along the channel centre and pulled away from the channel top and bottom.

To quantify the intrinsic flow behaviour at a fixed finite Reynolds number, the cross-sectional area and perimeter of the particles are plotted in Fig. 4c,d, respectively. Surprisingly, for the side-pillar case, the cross-sectional area was increased as pillar number increased, whereas area of the centre-pillar case was decreased. This can be attributed to the fact that the inertial flow deformation is a 3D flow stretching phenomenon. The displaced volume should remain constant as fluid passes pillars, although the cross-sectional area does not necessarily remain constant. Note that the secondary flow deforms the fluid streams less as fluid passes additional pillars because the interactions between core fluids and secondary flow become saturated, leading to less changes in the flow cross-section (see Supplementary Fig. 1 for more details). The area difference between pillar layouts can be also described by the fact that secondary flow enhances the diffusion of photoinitiator into the sheath fluid. Inertial flow deformation can be considered as a flow folding process; therefore, larger deformation requires relatively shorter length and time to diffuse. We also introduced a characteristic length defined by the cross-sectional area divided by the perimeter, as shown in Fig. 4e. The particles created with two different pillar configurations (side and center pillars) showed similar trends. As both pillar configurations were tested under identical flow conditions/pillar specification (for example, diameter), but with different lateral pillar-positions, both cases were expected to experience similar flow deformation processes.

### Flow shaping via *Re* number

**Figure 5:**
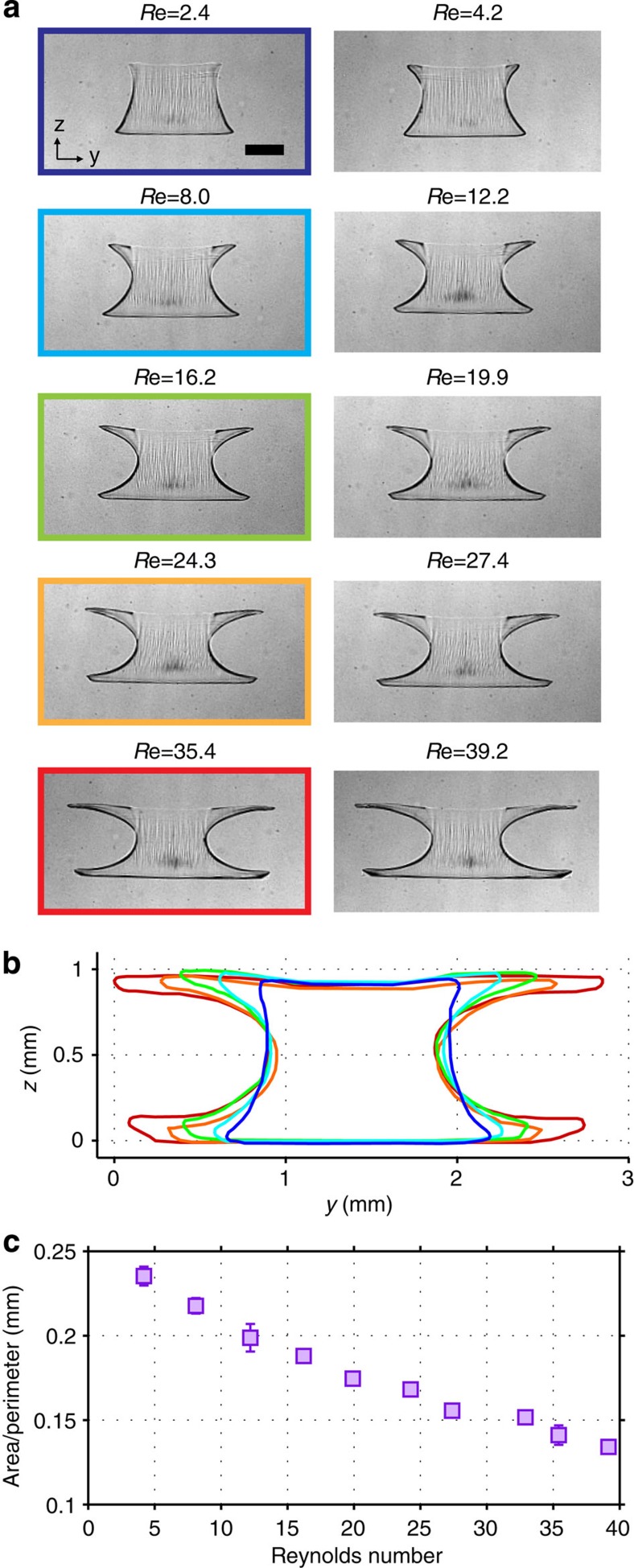
Flow deformation dependence on Reynolds number. (**a**) Using a side-pillar channel, particles are created after passing the third pillar (P3) with different Reynolds numbers. (**b**) Perimeter of particles from (**a**) plotted with a common origin. (**c**) Deformation characteristic length (particle area/particle perimeter) plot as a function of Reynolds number. Error bars represent one standard deviation from three different experimental setups. Scale bar represents 500 μm.

Previously, changes in pillar numbers and pillar configurations were investigated to exert various secondary flow patterns, but as an alternative flow condition, the Reynolds number can be varied to manipulate flow deformation. In general, a higher Reynolds number indicates higher inertia (that is, more interactions between fluid and structures), leading to an increase of inertial deformation. To demonstrate this, we conducted the particle polymerization in the same manner as shown in Fig. 4 using a thin-slit photomask. Figure 5 shows particles all created at the same location (P3: after passing the third pair of side pillars). At low Reynolds numbers, the particle generated at P3 should remain similar to the particle created at P0. However, slight asymmetry (wider along the particle bottom than that of top) was noticed because the core fluid contained additional photoinitiator (DMPA), resulting in a denser fluid. For the higher Reynolds number cases, faster flow rates allows the core fluid less time to settle towards the channel bottom due to the density difference before reaching P3, allowing for more symmetric-shaped particles. From images taken of the particles, the coordinates of the particle outlines were found, and then plotted using a common origin (Fig. 5b) for a visual comparison of particle shape based on Reynolds number. As expected, higher flow rate (that is, higher inertia) caused increased flow deformation. The deformation characteristic length (particle area/particle perimeter) was also plotted for each Reynolds number case (Fig. 5c), showing a trend similar to pillars 0 to 6 from Fig. 4e.

### Light pattern dependence

**Figure 6:**
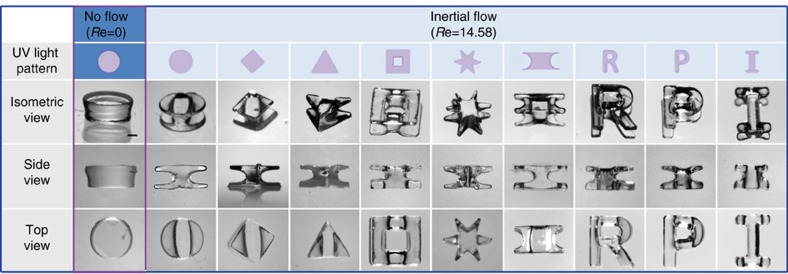
Light pattern dependence. Creation of 3D-shaped particles using different photomask patterns, but using identical flow conditions. The 3D-shaped particles are polymerized at P16 using a side-pillar channel. As a control, a 2D particle was first created in a static flow condition (*Re*=0), shown in the left column. Scale bar represents 500 μm.

Along with flow conditions, the polymerized particle shapes are also determined by light conditions. As a control, we first polymerized particles under static flow conditions, analogous to SFL^28^,^29^ and OFML^30^,^31^. A circular light pattern was used to polymerize, resulting in a 2D cylindrical-shaped particle (see Fig. 6; left column). Note that the top of the cylinder was slightly wider due to diffraction of the UV light in the axial direction. For the inertial cases (*Re*=14.58), all experiments were conducted under fixed flow conditions (that is, same pillar number, configuration and identical *Re*). We used a side-pillar channel, as seen in Fig. 4a, and light was illuminated to an identical polymerization spot (P16; after passing 16 pairs of side pillars) using various photomask patterns. As shown in Fig. 6, a variety of 3D-shaped particles that cannot be created from typical flow lithography, SFL or OFML techniques are presented (see Supplementary Movie 4). As the identical flow conditions were applied, the fabricated particles possessed identical cross-sectional (‘I') shapes; however, a larger set of shapes is available by varying flow circumstances (see the following section for more details).

### Complex shape fabrication

**Figure 7:**
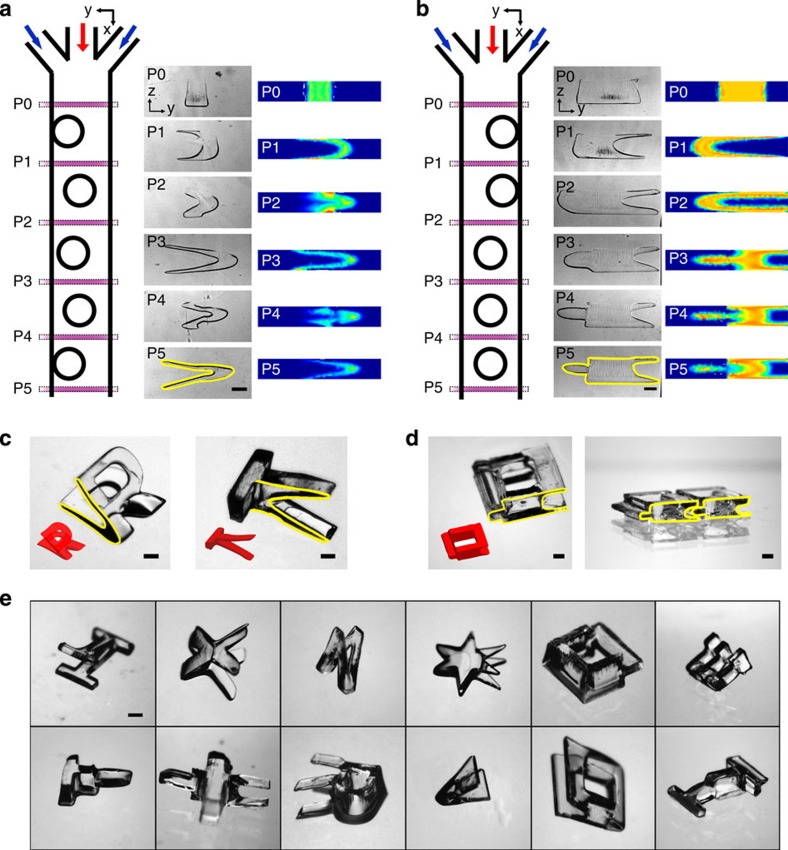
Complex shaped particle creation. Variation of the lateral position of sequential pillars allows for richer flow transformations to create more complex shapes including (**a**) a ‘V'-shaped and (**b**) arbitrary-shaped flow cross-section. (**c**) Experimental and predicted 3D particle shapes created with ‘V' cross-section using ‘R'- and ‘T'-shaped light patterns. (**d**) Particles created from **b** for direct- and self-assembly applications. (**e**) Assortment of 3D particles using various light patterns and flow cross-sections from **a** and **b**. Scale bars represent 500 μm.

Complex pillar configurations can be used to exert exotic but deterministic and predictable flow deformation patterns that can be combined with chosen light patterns. As mentioned earlier, varying the lateral placement of pillars within a channel changes the shape, number and location of secondary flow vortices^43^,^44^. In addition, even with the same channel, complex flow cross-sectional shapes can be achieved with higher Reynolds numbers^43^,^44^, although higher *Re* operation is not desired because of potential channel damage (for example, leakage) from larger pressure drops and increased time required to stop the flow before UV illumination, inducing unwanted diffusion. We tested various pillar combinations, and two representative cases are presented in Fig. 7. For instance, a ‘V' shape cross-section was created (see Fig. 7a) and experimentally fabricated shapes showed very good agreement with the numerical prediction. Again, a thin-slit-shaped photomask was used for Fig. 7a,b to capture the evolution of flow deformation, but by using other light patterns, various complex 3D-shaped particles were created, as can be seen in Fig. 7c–e (see Fig. 1a for more examples). By combining different pillar configurations with various Reynolds numbers and UV light patterns, a large set of complex shapes are available.

## Discussion

The presented optofluidic fabrication provides many fundamental advantages when compared to existing 3D particle generation methods. Flow shaping allows for a smooth particle side-profile with high *z*-directional resolution in a single UV polymerization step, eliminating the need for slow and low-resolution layer-by-layer–based processes. Through optofluidic fabrication, we also demonstrate the creation of inverse structures (for example, ‘I' cross-sectional shapes) without the need for internal physical support, which most rapid prototyping methods require. Moreover, as the technology only utilizes passive elements such as pillars or columns to shape the flow, it offers a simple production scheme well-suited for full automation.

The current throughput of our optofluidic fabrication system is 3,600 particles per hour (using ten simultaneously polymerized particles per UV exposure: Supplementary Movie 5 and Supplementary Note 1), although a much higher throughput is possible via off-microscope particle production. An off-microscope setup permits massive channel parallelization and wide-field UV light exposure where we can expect a throughput of >700,000 particles per hour (see Supplementary Note 1 and Supplementary Fig. 2). Note that this throughput can be increased by shortening the cycle period. For instance, a more powerful UV light source with optical elements with less UV absorption will decrease the exposure time needed for particle polymerization, leading to a faster overall process. More rapid exposure would also likely improve microscale feature resolution because it would reduce diffusive blurring of photoinitiator.

It should be mentioned that in addition to scaling down to micrometre scales, scaling up of the particle size is also possible. Currently, particles range between 1 and 2 mm in size with the tested conditions. However, optofluidic fabrication can be scaled up to centimetre scales or larger by inducing identical flow behaviour (that is, matching Reynolds number) in large-scale channels along with an advanced light setup (for example, stronger UV light to provide sufficient energy for crosslinking).

Another fundamental advantage of optofluidic particle fabrication is the ability to produce functional particles. For instance, similar to particles seen in Fig. 7d, direct- or self-assembling particles can be made possible^31^ by creating particle shapes that can latch onto one another in two or even three dimensions. Anisotropic particles can be created by encapsulating heterogeneous elements such as metal or magnetic beads where we can expect unique behaviours. Functional particles can also be created by taking advantage of diffusion between fluid streams, which can be useful for optics, such as graded index lenses or fibres^47^. Another promising direct application from optofluidic fabrication can be found in tissue engineering. As cells grow and interact with their environments in a 3D manner, the ability to create 3D structures with internal pore networks is crucial^48^. Current methods are limited at forming internal vascularized tissue constructs on the scale of tens of micrometres^49^, but optofluidic fabrication has the ability to create hydrogel structures with internal networks at these scales (for example, swap the core and sheath fluids in Fig. 4b), and without the aid of physical supports.

Material selection diversity is another merit of the reported technology. The presented optofluidic fabrication creates polymeric particles based on cross-linking of PEG-DA with DMPA, but the overall particle formation principle is not limited to the tested material. Any light-polymerizable material with appropriate properties (for example, density and viscosity for flow shaping) can be used. We are currently testing other precursor candidates such as thiolene-based polymers to achieve variable polymerization duration and particle mechanical properties.

Particle shape diversity and dynamic tunability from optofluidic fabrication should also be highlighted. Nearly, an infinite number of different particle shapes can be created by varying flow and light conditions. Integration with a digital micro-mirror device^31^ will allow on-the-fly changes of particle shapes from a single channel along with variable flow conditions.

## Methods

### Channel fabrication

Microfluidic channels were created from PDMS using standard soft lithography techniques^50^. Replicas were made by pouring Sylgard 184 Silicone Elastomer (Dow Corning Corporation) over a 3D printed plastic master (Purple Porcupine). Glass substrates were coated with a thin PDMS layer and were treated along with replicas by a plasma cleaner (Harrick Plasma). Replicas and substrates were then bonded together to form channels. Channels were incubated overnight in a convection oven (VWR) at 65 °C.

### Precursor fluids

Inert fluid streams (sheath fluid) consisted of PEG-DA, *M*_n_=250 (Sigma-Aldrich), whereas photosensitive fluid streams (core fluid) consisted of PEG-DA with 5 wt% DMPA (Sigma-Aldrich). Fifteen micrometre fluorescent polystyrene tracers (Thermo Fisher Scientific) were added to visualize fluid flow. A programmable pressure controller was used to control and pump the fluid flow.

### Imaging and UV illumination

Experiments were performed on a Zeiss Axio Ovserver.A1 (Carl Zeiss) inverted microscope and imaged using a Phantom high speed camera (Vision Research, Inc.). UV light at 365 nm was directed through the microscope objectives along with a mechanical shutter to control UV light exposure time. A photomask was placed in the microscope field stop to create patterned UV light.

## Author contributions

K.S.P., D.D.C. and A.J.C. designed the research. K.S.P. and A.J.C. designed the device and experimental setup. K.S.P. performed the experiments and numerical studies. K.S.P., D.D.C. and A.J.C. analysed the data and wrote the manuscript.

## Additional information

**How to cite this article:** Paulsen, K.S. *et al*. Optofluidic fabrication for 3D-shaped particles. *Nat. Commun.* 6:6976 doi: 10.1038/ncomms7976 (2015).

## Supplementary Material

Supplementary Information Figures 1-2 and Supplementary Note 1

Supplementary Movie 1 Optofluidic fabrication of X-shaped particle. Using a side-pillar channel with *Re* = 14.58, ‘X' patterned UV light is exposed to the channel after pillar P16 to create a complex shaped particle. The particle displays an ‘X' shape from the top view and an ‘I' shaped cross section.

Supplementary Movie 2 Stokes flow vs. inertial flow. A side pillar channel was used to visualize the deformation of core fluid for a Stokes flow (*Re* = 0.003) and inertial flow case (*Re* = 14.58). For both cases, the core fluid was dyed with the fluorescent dye Rhodamine B. For the Stokes flow case, a slight widening of the core fluid is seen downstream due to diffusion of the fluorescent dye into the sheath fluid. Significant widening of the core fluid is seen for the inertial flow case.

Supplementary Movie 3 UV Polymerization to reveal flow cross section. A photomask with a thin slit was used to polymerize a particle at P6 for a side-pillar channel and P7 for the center-pillar channel. The cross sectional shape of the particles revealed the flow cross sectional shape.

Supplementary Movie 4 Optofluidic fabrication of numerous 3D particles. For a side-pillar channel with *Re* = 14.58, different UV light patterns were used to create particles with an ‘I' shaped cross section, but with different top-view shapes.

Supplementary Movie 5 Multi-particle generation with a single UV exposure. For a side-pillar channel with *Re* = 14.58, five fluid streams were used with three inert streams and two photosensitive streams. Ten particles are simultaneously polymerized with a single UV exposure step, and this is repeated with a 10 second cycle time.
