# Supplementary material for: Optofluidic fabrication for 3D-shaped particles
Source: Nat Commun. 2015 Apr 23;6:6976. doi: 10.1038/ncomms7976 (PMC4421806; doi:10.1038/ncomms7976)
Supplement: Supplementary Information — Figures 1-2 and Supplementary Note 1 [file ncomms7976-s1.pdf]

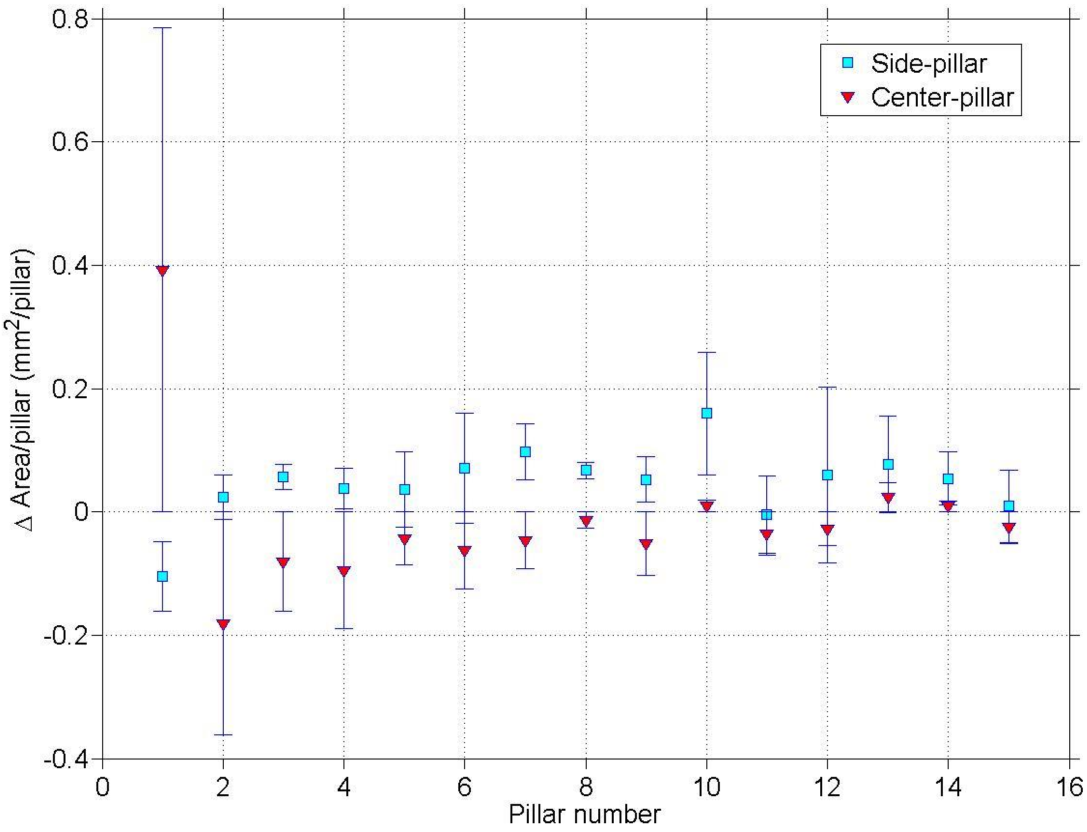

Supplementary Figure 1: Change in particle cross sectional area per pillar. As fluid passes sequential pillars, each additional pillar results in a smaller change in flow cross sectional area, converging to zero because the interactions between core fluid and secondary flow saturate. Error bars represent one standard deviation from the mean (*n* = 3; from different experimental setups).

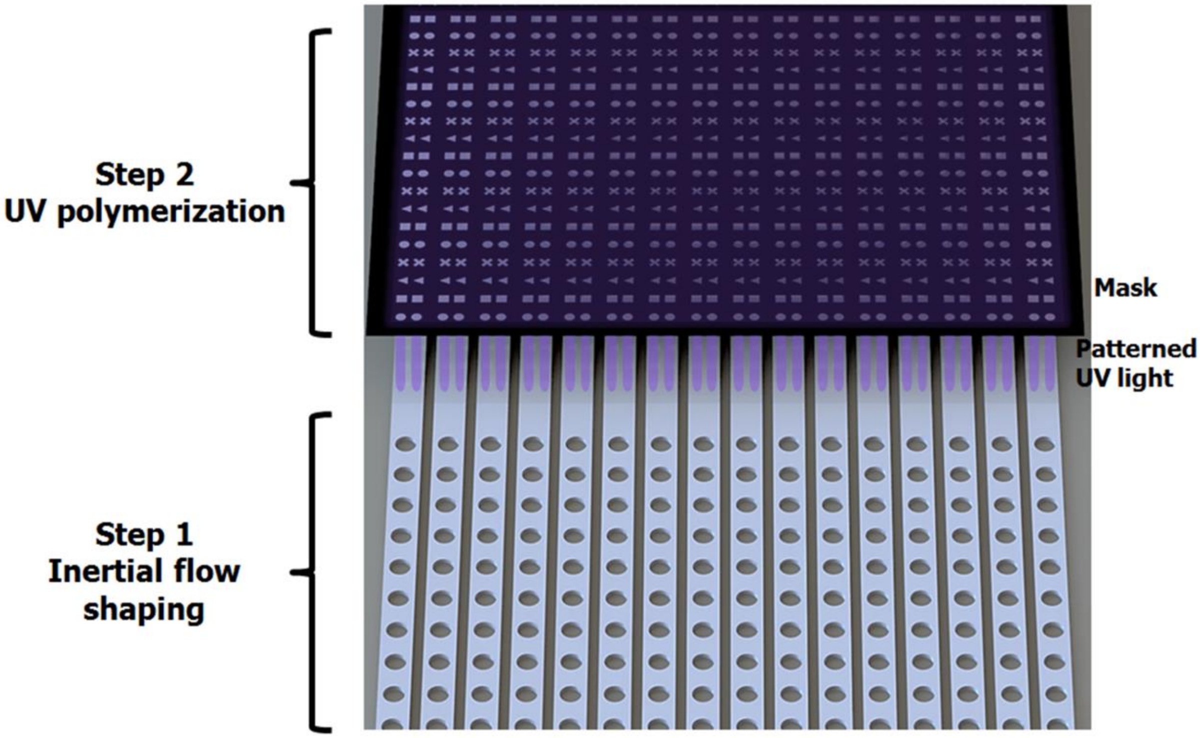

Supplementary Figure 2: Off-microscope parallelization for high-throughput particle generation. Using a larger UV light source, multiple channels can be used in parallel to increase throughput. In addition to polymerizing two rows of particles in the channel lateral direction (see Supplementary Movie 5), particles can be polymerized along the primary flow direction. With this off-microscope parallelized setup, a throughput of approximately 0.7 million particles per hour can be achieved (see Supplementary Note 1 for calculation).

[Supplementary Note 1]

Throughput Calculations

On-microscope throughput

For a side-pillar channel with *Re* = 14.58, five fluid streams were used with three inert streams and two photosensitive streams (see Movie S5). A photomask was used which illuminated ten different patterned light shapes along the channel primary flow direction. After passing pillars, the flow was stopped, and ten particles were polymerized simultaneously in a single UV exposure step. The UV exposure steps could be repeated with a 10 cycle period, resulting in a throughput of 3,600 particles per hour:

Particles polymerized per 1 UV exposure:

$$\left(\frac{10\ particles}{1\ UVexposure}\right)\left(\frac{1\ UVexposure}{10\ seconds}\right)\left(\frac{3{,}600\ seconds}{1\ hour}\right) = \textbf{3,600 particles/hour}$$

Off-microscope throughput

By taking optofluidic fabrication off of the microscope, parallelized channels with a larger UV light source can be used to move optofluidic fabrication towards a high-throughput process (see Supplementary Fig. 2). By using a 5 inch x 5 inch collimated UV light source (similar to a contact aligner for microfabrication), 16 of the presented channels may be used in parallel. Channels have a width W_c = 6 mm and maintain a channel-to-channel spacing of S_c = 2 mm. After passing pillars to shape the flow, the channels consist of a straight section of length 130 mm, void of any geometric obstructions, to utilize the full 5 inch x 5 inch UV light. Particles will be polymerized along this straight section. As demonstrated in Supplementary Movie 5, two rows of particles will be polymerized in the channel lateral direction. Particles will have a width W_p = 1.5 mm, and a particle-to-particle spacing S_p = 0.5 mm along the length of the channel.

Particles polymerized in 1 channel:

5 in x 5 in UV light ⇒ Possible polymerization length L = 127 mm

Particles polymerized per 1 UV exposure in 1 channel:

$$(N_{rows})\frac{L}{W_p+W_s} = 2\cdot\frac{127\ mm}{(1.5mm+0.5mm)/particle} = 2\cdot 63.5 \approx 126\ particles$$

$$\approx 126\ \frac{particles}{1\ UV\ exposure\cdot channel}$$

Note: The factor of 2 arises from 2 rows of particles being polymerized in the lateral direction of a single channel.

Particles polymerized per 1 UV exposure:

$$(16\ channels)\left(\frac{126\ particles}{1\ UVexposure\cdot channel}\right)\left(\frac{1\ UVexposure}{10\ seconds}\right)\left(\frac{3{,}600\ seconds}{1\ hour}\right) = \textbf{725,760 particles/hour}$$
